# Dependence of paracentric inversion rate on tract length

**DOI:** 10.1186/1471-2105-8-115

**Published:** 2007-04-03

**Authors:** Thomas L York, Rick Durrett, Rasmus Nielsen

**Affiliations:** 1 Department of Biological Statistics and Computational Biology, Cornell University, Ithaca, USA; 2 Department of Mathematics, Cornell University, Ithaca, USA; 3 Center for Bioinformatics, University of Copenhagen, Copenhagen, Denmark

## Abstract

**Background:**

We develop a Bayesian method based on MCMC for estimating the relative rates of pericentric and paracentric inversions from marker data from two species. The method also allows estimation of the distribution of inversion tract lengths.

**Results:**

We apply the method to data from *Drosophila melanogaster *and *D. yakuba*. We find that pericentric inversions occur at a much lower rate compared to paracentric inversions. The average paracentric inversion tract length is approx. 4.8 Mb with small inversions being more frequent than large inversions.

If the two breakpoints defining a paracentric inversion tract are uniformly and independently distributed over chromosome arms there will be more short tract-length inversions than long; we find an even greater preponderance of short tract lengths than this would predict. Thus there appears to be a correlation between the positions of breakpoints which favors shorter tract lengths.

**Conclusion:**

The method developed in this paper provides the first statistical estimator for estimating the distribution of inversion tract lengths from marker data. Application of this method for a number of data sets may help elucidate the relationship between the length of an inversion and the chance that it will get accepted.

## Background

Reconstructing the history of inversions and/or translocations separating two chromosomes or genomes is a classical problem in computational biology dating back as far as early work by the pioneers of genetic research from the 1930's (eg. [1]). In many applications, this problem has been treated as a problem of finding the minimum number of events required in the evolutionary history of the two genomes. The computational problem involved is known as sorting by reversal (e.g. [2-4]). An alternative approach is to estimate the number of events using statistical estimators that take into account that more inversions (and translocations) may have occurred than the minimum possible number. Larget et al. [5] and York et al. [6] have developed Bayesian methods based on Markov Chain Monte Carlo (MCMC) for estimating the history of inversions separating two chromosomes. The following description is based on the method of York et al. [6]. In brief, a Markov chain is established that has, as its stationary distribution, the posterior distribution of inversion paths (possible histories of inversions).

The likelihood function is calculated assuming inversions occur according to a Poisson process and assuming a uniform prior over all possible inversions paths of a fixed length. The inversion path is then represented explicitly in the computer memory and updates are proposed according to a proposal kernel, allowing exploration of the posterior distribution. The update kernel is guided by the parsimony distance computed from the breakpoint graphs developed for solving the sorting by reversal problem [4,7]. Using the parsimony distance to guide updates greatly increases convergence rates of the Markov chain. Point estimates of the number of inversions, with associated measures of statistical confidence are then obtained from the posterior distribution. The method of [6] was extended in [8] to the case of multiple chromosomes differing by an unknown number of translocations and inversions. Similarly, [9] extends this type of approach to rearrangements due to transpositions and inverted transpositions in addition to inversions. The advantage of these Bayesian approaches is that they use all of the information in the marker data to obtain a statistical estimate of the number of inversions and translocations. However, so far these approaches have assumed that a long chromosomal segment is as likely to be inverted as a short one, and have lumped together pericentric and paracentric inversions rather than distinguishing between them. Pericentric inversions appear to be rarer than paracentric ones, and there is evidence for a length-dependent effect also [10,11], with selection related to recombination in the inverted region of inversion heterozygotes as a possible cause. Another simplification made hitherto is that only the order of markers in a set (and their orientations, in the case of signed data) has been used, so no account is taken of the uneven spacing of the markers. The objective of this paper is to modify the previous methods to take into account these factors. This will allow us to estimate the relative frequency of pericentric and paracentric inversions and to estimate the distribution of inversion tract lengths. We apply the new method to genomic data from *D. melanogaster *and *D. yakuba*.

The assumption of tract-length independence is relaxed also in [12], which considers the problem of finding the optimal inversion path when the cost of an inversion depends on its tract-length; they do not address determining that dependence from data.

## Results and Discussion

### Results

**Figure 1:**
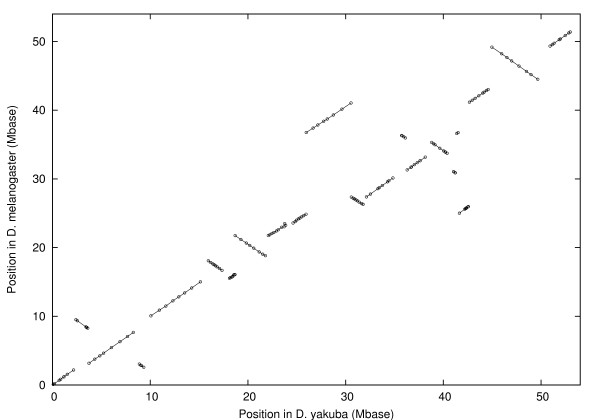
Position in *D. melanogaster *vs. position in *D. yakuba *for chains after filtering, chromosome 3. Lines show blocks.

We have analyzed a set of 388 markers on the three major chromosomes, 2, 3 and X, using distance information from *D. yakuba *but only marker order information from *D. melanogaster*. The positions of the centromeres are also used. For chromosome 3 we found 163 markers in 23 conserved blocks as shown in figure 1. For the purposes of finding the inversion distance between these two marker arrangements this may be represented as the following signed permutation:

1, -4, 3, -2, 5, -7, 6, -8, 9, -10, 11, 20, -13, 14, -18, 16, -17, -15, 19, 12, 21, -22, 23.

**Figure 2:**
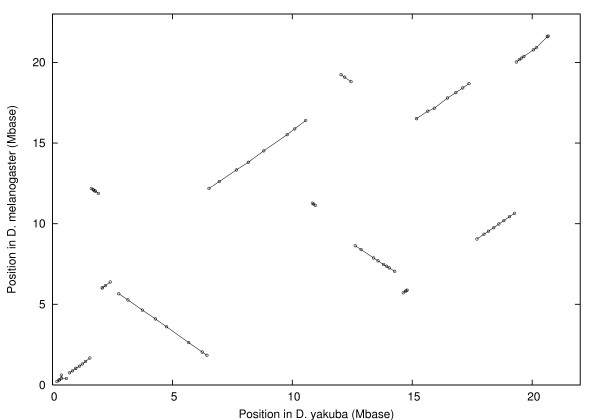
Position in *D. melanogaster *vs. position in *D. yakuba *for chains after filtering, chromosome X. Lines show blocks.

The inversion distance is 13. The centromere is between blocks 10 and 11. For chromosome X we found 84 markers in 14 blocks as shown in figure 2. The signed permutation is:

1, -2, 3, -10, -4, 11, -9, -13, -7, 5, 12, 8, 14.

**Figure 3:**
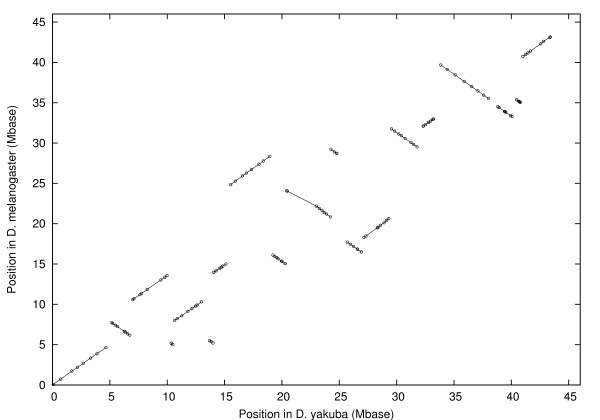
Position in *D. melanogaster *vs. position in *D. yakuba *for chains after filtering, chromosome 2. Lines show blocks.

The inversion distance is 7. The centromere is between block 14 and the chromosome end. Similarly for chromosome 2 we found 141 markers in 19 blocks as shown in figure 3. The signed permutation is:

1, -4, 6, -2, 5, -3, 7, 12, -8, -11, -13, -9, 10, -14, 15, -18, -16, -17, 19.

The inversion distance is 11 including one pericentric inversion. The centromere is in the middle of block 11. Thus, it takes at least 31 inversions, including at least one pericentric inversion, to turn the *D. yakuba *marker arrangement on chromosomes 2, 3 and X into the *D. melanogaster *arrangement.

**Figure 4:**
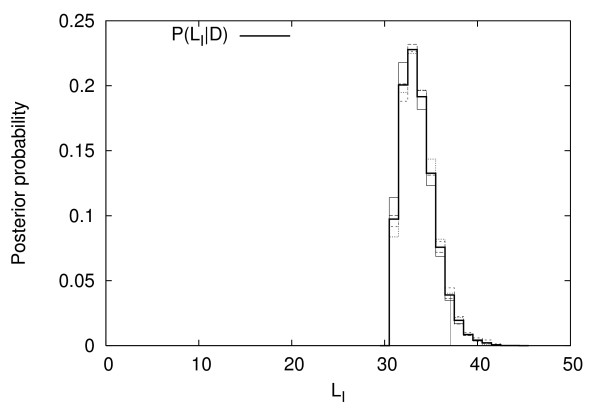
Posterior distribution of the number of inversions *L*_*I*_. Vertical lines indicate the 95% credible interval.

A run of 1.1 × 10^6 ^updates was performed, taking 90 cpu hours on a 1.8 GHz Athlon processor. The first 1.1 × 10^5 ^updates were discarded as burn-in. Figures 4 through 6 show histograms of quantities of interest using the remainder of the MCMC output; agreement among the four replicate chains (shown with dotted and dashed lines) is very good, indicating good MCMC convergence.

**Figure 5:**
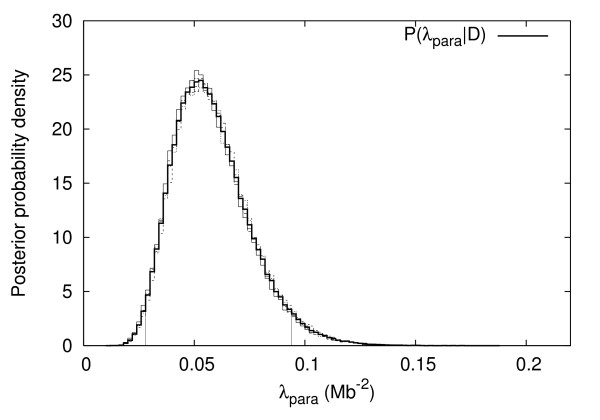
Posterior distribution of *λ*_*pa*_. Vertical lines indicate the 95% credible interval.

**Figure 6:**
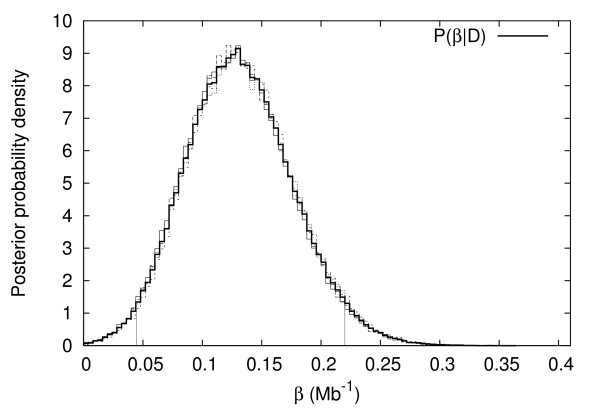
Posterior distribution of exponential tract-length dependence parameter *β*. Vertical lines indicate the 95% credible interval.

Table 1 lists 95% credible intervals and maximum a posteriori (MAP) estimates of the number of parametric inversions, *L*_*pa*_, the rate parameters for paracentric, and pericentric, *λ*_*pa *_and *λ*_*pe*_, and the parameter *β *which describes the strength of the tract-length dependent effect in our model. These parameters are more fully defined in the methods section.

**Table 1:** 

	95% credible interval	MAP estimate	units
*L*_*pa*_	[30,36]	32	
*λ*_*pa*_	[0.028, 0.094]	0.053	*Mb*^-2^
*λ*_*pe*_	[0, 0.0041]	7.5 × 10^-4^	*Mb*^-2^
*β*	[0.044, 0.22]	0.13	*Mb*^-1^

From figure 4 it is clear that the number of inversions is compatible with the parsimony estimate of 31 inversions. However, the most likely number of inversions is 33. The credible interval (Table 1) excludes more than 37 inversions at the 95% level. The 95% credible interval for the rate parameter *λ*_*pa *_is [0.028, 0.094] *Mb*^-2^.

A minimum of one pericentric inversion (on chromosome 2) is needed to rearrange the markers, and the posterior probability of > 1 pericentric inversions is < 1 × 10^-4^. The pericentric rate parameter, *λ*_*pe*_, is correspondingly small, with a MAP estimate of 7.5 × 10^-4 ^*Mb*^-2^.

The MAP estimate of the tract length dependence parameter (*β*) is 0.130 *Mb*^-1 ^with a 95% credible interval of [0.044, 0.22] *Mb*^-1 ^(Table 1 and Figure 6). Using (6), and the chromosome arm lengths (20.7, 22.3, 21.2, 24.2 and 28.7 Mb), we find that *β*_*MAP *_= 0.130 *Mb*^-1 ^corresponds to a mean tract length of 4.8 *Mb*, compared with 8.1 *Mb *assuming *β *= 0.

The fact that *β *is positive, and that values of *β *close to zero receive very little support shows that small tract lengths are favored over large tract lengths.

**Figure 7:**
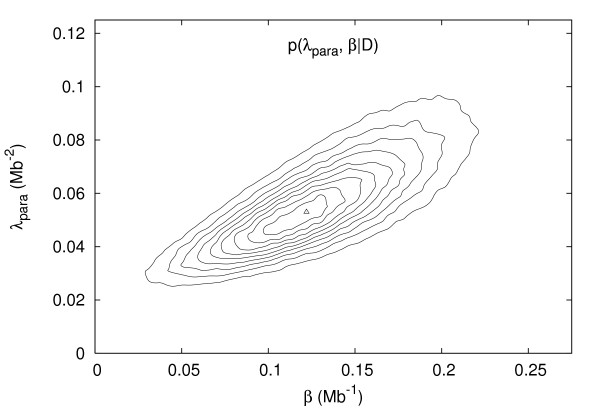
Posterior joint distribution of *λ*_*pa *_and *β*. The triangle marks the mode.

Figure 7 shows the posterior joint distribution of *β *and *λ*_*pa*_, together with the corresponding MAP estimate (*β*, *λ*_*pa*_)_*MAP *_= (0.122 *Mb*^-1^, 0.053 *Mb*^-2^). The observed positive correlation between the two parameters is not surprising. The rate of inversions of tract length *τ *is proportional to *λ*_*pa*_*e*^-*βτ*^(ℓ - *τ*), which for *λ*_*pa *_> 0 and 0 <*τ *≤ ℓ is a decreasing function of *β*. Unless increasing *β *(favoring short tract lengths more) allows the observed rearrangement to be accomplished with fewer inversions, then *λ*_*pa *_must increase as *β *does.

## Discussion

One drawback of the current method is that, as is the case for many other MCMC methods, it is computationally slow. Nonetheless, the speed of the program is not so slow that it is prohibitive, as illustrated in the analysis of the *Drosophila *data. The existing program should be able to handle somewhat larger data sets (up to perhaps 100 blocks and 800 markers) by some combination of running longer, running replicate chains on separate processors, and, in some cases breaking a multi-chromosome data set down into individual chromosomes or arms and analyzing each one separately. To go beyond that would require substantial work to improve the algorithm. A related issue is the resolution at which we can analyze genome rearrangements. In addition to the increased computational burden of analyzing more and shorter blocks, the assumption implicit in our method that the observed rearrangement is due solely to inversions (and translocations) becomes more problematic for smaller scale rearrangements. The method is, therefore, more suitable for making statements regarding inversions occurring at the scale of hundreds of kilobases or megabases than at the scale of a few kilobases. Nonetheless, with several blocks less than 200 kilobases long in our data, and 5 chromosome arms totaling 117 megabases, we are apparently sensitive to inversion tract-lengths down to about 1% of the chromosome arm length.

## Conclusion

The method developed in this paper provides the first statistical estimator for estimating the distribution of inversion tract lengths from marker data. Application of this method to a number of data sets may help elucidate the relationship between the length of an inversion and the chance that it will get accepted.

## Methods

### The model

#### Using marker order information only

Previously [6,8] we have considered models of rearrangements of *M *markers on *C *chromosomes, in which only the order of the markers is used. Two arrangements of a set of markers are considered to be the same if and only if every pair of markers adjacent in one arrangement is also adjacent in the other, and every marker adjacent to a chromosome end in one arrangement is adjacent to a chromosome end in the other. In the case of a single chromosome the markers divide it into *M *+ 1 segments and we can distinguish *N*_*I *_= *M*(*M *+ 1)/2 inversions corresponding to unordered pairs of distinct segments. Assuming a Poisson process with rate Λ, and assuming the *N*_*I *_inversions to be equiprobable, the probability of a particular path *X *consisting of *L *inversions is:

P(X|Λ)=e−ΛΛLL!NI-L=e−ΛL!λL
 MathType@MTEF@5@5@+=feaafiart1ev1aaatCvAUfKttLearuWrP9MDH5MBPbIqV92AaeXatLxBI9gBamXvP5wqSXMqHnxAJn0BKvguHDwzZbqegyvzYrwyUfgarqqtubsr4rNCHbGeaGqiA8vkIkVAFgIELiFeLkFeLk=iY=Hhbbf9v8qqaqFr0xc9pk0xbba9q8WqFfeaY=biLkVcLq=JHqVepeea0=as0db9vqpepesP0xe9Fve9Fve9GapdbaqaaeGacaGaaiaabeqaamqadiabaaGcbaGaemiuaaLaeiikaGIaemiwaGLaeiiFaWNaeu4MdWKaeiykaKIaeyypa0ZaaSaaaeaacqWGLbqzdaahaaWcbeqaaiabgkHiTiabfU5ambaakiabfU5amnaaCaaaleqabaGaemitaWeaaaGcbaGaemitaWKaeiyiaecaaiabd6eaonaaDaaaleaacqqGjbqsaeaacqqGGaaicqqGTaqlcqqGmbataaGccqGH9aqpdaWcaaqaaiabdwgaLnaaCaaaleqabaGaeyOeI0Iaeu4MdWeaaaGcbaGaemitaWKaeiyiaecaaGGaciab=T7aSnaaCaaaleqabaGaemitaWeaaaaa@5CE0@

where *λ *= Λ/*N*_*I*_. The posterior probability density is then

p(X,λ|D)∝P(D|X)e−ΛL!λLp(λ).
 MathType@MTEF@5@5@+=feaafiart1ev1aaatCvAUfKttLearuWrP9MDH5MBPbIqV92AaeXatLxBI9gBaebbnrfifHhDYfgasaacH8akY=wiFfYdH8Gipec8Eeeu0xXdbba9frFj0=OqFfea0dXdd9vqai=hGuQ8kuc9pgc9s8qqaq=dirpe0xb9q8qiLsFr0=vr0=vr0dc8meaabaqaciaacaGaaeqabaqabeGadaaakeaacqWGWbaCcqGGOaakcqWGybawcqGGSaaliiGacqWF7oaBcqGG8baFcqWGebarcqGGPaqkcqGHDisTcqWGqbaucqGGOaakcqWGebarcqGG8baFcqWGybawcqGGPaqkdaWcaaqaaiabdwgaLnaaCaaaleqabaGaeyOeI0Iaeu4MdWeaaaGcbaGaemitaWKaeiyiaecaaiab=T7aSnaaCaaaleqabaGaemitaWeaaOGaemiCaaNaeiikaGIae83UdWMaeiykaKIaeiOla4caaa@4CED@

The prior *p*(*λ*) is taken to be uniform between 0 and *λ*_*max*_, and zero elsewhere. The data in this case are the marker orders *D*_1 _and *D*_2 _observed in two taxa. A path *X *starting at *D*_1 _either ends up at *D*_2_, in which case *P*(*D*|*X*) = 1, or it ends up at some order other than *D*_2_, in which case *P*(*D*|*X*) = 0.

We construct an initial path by starting with *D*_1_, and performing inversions and translocations until *D*_2 _is obtained. Using the Hannenhalli-Pevzner breakpoint graph theory of sorting by reversal (i.e. by inversions) [4,7], which has been used in all the MCMC sampling approaches to the inversion problem [5,6,9,8], we preferentially choose rearrangements that lead to short paths. Proposed updates are constructed by choosing two points along the existing path and constructing a path between them in the same way, thus guaranteeing *P*(*D*|*X*) = 1.

Starting from a particular marker order there are *N*_*I *_distinct inversions, each occurring with rate *λ*, i.e., the probability of a particular inversion occurring in a short time *t *is *λt *where time is scaled such that the whole rearrangement process takes unit time.

In order to handle multiple chromosomes and translocations [8], we require distinct parameters *λ*_*I *_and *λ*_*T *_for the rates of inversions and translocations respectively. For each arrangement of markers there will be some number of inversions *N*_*I *_and some number of translocations *N*_*T*_; both of these depend on how many markers are on the various chromosomes, and therefore can change along a path. For this reason we now uniformize the process by defining a total event rate Λ(*λ*_*I*_, *λ*_*T*_) which is guaranteed to be at least as great as the sum of the total inversion and total translocation rates,

Λ(*λ*_*I*_, *λ*_*T*_) > Λ_*real *_≡ Λ_*I *_+ Λ_*T *_≡ *N*_*I*_*λ*_*I *_+ *N*_*T*_*λ*_*T*_, with "dummy" rearrangments (which have no effect on the genome) occurring with rate *λ*_*d *_= Λ - Λ_*real*_. Now Λ(*λ*_*I*_, *λ*_*T*_) is fixed along the path and we may write

P(X|λI,λT)=e−ΛL!λILIλTLT∏kλdk
 MathType@MTEF@5@5@+=feaafiart1ev1aaatCvAUfKttLearuWrP9MDH5MBPbIqV92AaeXatLxBI9gBaebbnrfifHhDYfgasaacH8akY=wiFfYdH8Gipec8Eeeu0xXdbba9frFj0=OqFfea0dXdd9vqai=hGuQ8kuc9pgc9s8qqaq=dirpe0xb9q8qiLsFr0=vr0=vr0dc8meaabaqaciaacaGaaeqabaqabeGadaaakeaacqWGqbaucqGGOaakcqWGybawcqGG8baFiiGacqWF7oaBdaWgaaWcbaGaemysaKeabeaakiabcYcaSiab=T7aSnaaBaaaleaacqWGubavaeqaaOGaeiykaKIaeyypa0ZaaSaaaeaacqWGLbqzdaahaaWcbeqaaiabgkHiTiabfU5ambaaaOqaaiabdYeamjabcgcaHaaacqWF7oaBdaqhaaWcbaGaemysaKeabaGaemitaW0aaSbaaWqaaiabdMeajbqabaaaaOGae83UdW2aa0baaSqaaiabdsfaubqaaiabdYeamnaaBaaameaacqWGubavaeqaaaaakmaarafabaGae83UdW2aaSbaaSqaaiabdsgaKnaaBaaameaacqWGRbWAaeqaaaWcbeaaaeaacqWGRbWAaeqaniabg+Givdaaaa@5358@

where the product is over the dummy events on the path, indexed by *k*. Note that the path *X *here is a sequence of inversions, translocations and dummies.

#### Using distance information

Often, in addition to knowing the order of markers, we have some form of distance information, such as recombination distance or number of nucleotides between markers. We use this information by generating proposed paths which start from one of the genomes (call it genome 1) specified in the data, with not only the marker order being as specified, but also the distances. The distance information for genome 2 is ignored. A path is then constructed which has distance information at every step, but only the marker *order *at the end of the path is required to agree with genome 2. If distance information is available for both genomes, we can choose to use the distance information from either genome but not from both. We would like to be able to use the distance information from both genomes, where available, but we don't know how to construct a path which ends not only with a specified marker order, but also with (or close to) a specified set of inter-marker distances. This is particularly difficult because in reality the sum of these distances is not conserved.

Consider again for the moment a single chromosome, with *M *markers and length ℓ. When using only marker order information, we distinguished *N*_*I *_= *M*(*M *+ 1)/2 inversions and assumed equiprobability. Now, using distance information, an inversion is specified by the distances *x*_1 _and *x*_2 _of the breakpoints from one end of the chromosome, with (*x*_1_, *x*_2_) lying in the triangle 0 <*x*_1 _<*x*_2 _< ℓ, and we assume (for now) a uniform distribution over this region. The total rate of inversions is then *λ*_*I*_ℓ^2^/2 including inversions of segments containing zero markers. If we exclude these the rate is ΛI=λI(ℓ2−∑i=0Msi2)/2
 MathType@MTEF@5@5@+=feaafiart1ev1aaatCvAUfKttLearuWrP9MDH5MBPbIqV92AaeXatLxBI9gBaebbnrfifHhDYfgasaacH8akY=wiFfYdH8Gipec8Eeeu0xXdbba9frFj0=OqFfea0dXdd9vqai=hGuQ8kuc9pgc9s8qqaq=dirpe0xb9q8qiLsFr0=vr0=vr0dc8meaabaqaciaacaGaaeqabaqabeGadaaakeaacqqHBoatdaWgaaWcbaGaemysaKeabeaakiabg2da9GGaciab=T7aSnaaBaaaleaacqWGjbqsaeqaaOGaeiikaGIaeS4eHW2aaWbaaSqabeaacqaIYaGmaaGccqGHsisldaaeWaqaaiabdohaZnaaDaaaleaacqWGPbqAaeaacqaIYaGmaaaabaGaemyAaKMaeyypa0JaeGimaadabaGaemyta0eaniabggHiLdGccqGGPaqkcqGGVaWlcqaIYaGmaaa@44B1@ where the *s*_*i *_are distances separating adjacent markers. In the multiple chromosome case this becomes:

ΛI=λI2∑j=1C(ℓj2−∑i=0mjsij2)
 MathType@MTEF@5@5@+=feaafiart1ev1aaatCvAUfKttLearuWrP9MDH5MBPbIqV92AaeXatLxBI9gBaebbnrfifHhDYfgasaacH8akY=wiFfYdH8Gipec8Eeeu0xXdbba9frFj0=OqFfea0dXdd9vqai=hGuQ8kuc9pgc9s8qqaq=dirpe0xb9q8qiLsFr0=vr0=vr0dc8meaabaqaciaacaGaaeqabaqabeGadaaakeaacqqHBoatdaWgaaWcbaGaemysaKeabeaakiabg2da9maalaaabaacciGae83UdW2aaSbaaSqaaiabdMeajbqabaaakeaacqaIYaGmaaWaaabCaeaadaqadaqaaiabloriSnaaDaaaleaacqWGQbGAaeaacqaIYaGmaaGccqGHsisldaaeWbqaaiabdohaZnaaDaaaleaacqWGPbqAcqWGQbGAaeaacqaIYaGmaaaabaGaemyAaKMaeyypa0JaeGimaadabaGaemyBa02aaSbaaWqaaiabdQgaQbqabaaaniabggHiLdaakiaawIcacaGLPaaaaSqaaiabdQgaQjabg2da9iabigdaXaqaaiabdoeadbqdcqGHris5aaaa@4F1A@

where *j *now indexes chromosomes, and *m*_*j *_is the number of markers on chromosome *j*. In the corresponding expression for translocations:

ΛT=λTF∑j=1C∑k=j+1Cℓjℓk
 MathType@MTEF@5@5@+=feaafiart1ev1aaatCvAUfKttLearuWrP9MDH5MBPbIqV92AaeXatLxBI9gBaebbnrfifHhDYfgasaacH8akY=wiFfYdH8Gipec8Eeeu0xXdbba9frFj0=OqFfea0dXdd9vqai=hGuQ8kuc9pgc9s8qqaq=dirpe0xb9q8qiLsFr0=vr0=vr0dc8meaabaqaciaacaGaaeqabaqabeGadaaakeaacqqHBoatdaWgaaWcbaGaemivaqfabeaakiabg2da9GGaciab=T7aSnaaBaaaleaacqWGubavaeqaaOGaemOray0aaabCaeaadaaeWbqaaiabloriSnaaBaaaleaacqWGQbGAaeqaaOGaeS4eHW2aaSbaaSqaaiabdUgaRbqabaaabaGaem4AaSMaeyypa0JaemOAaOMaey4kaSIaeGymaedabaGaem4qameaniabggHiLdaaleaacqWGQbGAcqGH9aqpcqaIXaqmaeaacqWGdbWqa0GaeyyeIuoaaaa@49C3@

the factor *F *is the number of allowed translocations for each choice of breakpoints. After breaking two chromosomes into four pieces, there are 2 ways to put them back together (in addition to the initial configuration); if both of these are allowed then *F *= 2, but if we require every chromosome to always have exactly one centromere (as we will do later) then one of these is disallowed, and *F *= 1. Now instead of (3) we have

p(X|λI,λT)=e−ΛL!λILIλTLT∏kλdk,
 MathType@MTEF@5@5@+=feaafiart1ev1aaatCvAUfKttLearuWrP9MDH5MBPbIqV92AaeXatLxBI9gBaebbnrfifHhDYfgasaacH8akY=wiFfYdH8Gipec8Eeeu0xXdbba9frFj0=OqFfea0dXdd9vqai=hGuQ8kuc9pgc9s8qqaq=dirpe0xb9q8qiLsFr0=vr0=vr0dc8meaabaqaciaacaGaaeqabaqabeGadaaakeaacqWGWbaCcqGGOaakcqWGybawcqGG8baFiiGacqWF7oaBdaWgaaWcbaGaemysaKeabeaakiabcYcaSiab=T7aSnaaBaaaleaacqWGubavaeqaaOGaeiykaKIaeyypa0ZaaSaaaeaacqWGLbqzdaahaaWcbeqaaiabgkHiTiabfU5ambaaaOqaaiabdYeamjabcgcaHaaacqWF7oaBdaqhaaWcbaGaemysaKeabaGaemitaW0aaSbaaWqaaiabdMeajbqabaaaaOGae83UdW2aa0baaSqaaiabdsfaubqaaiabdYeamnaaBaaameaacqWGubavaeqaaaaakmaarafabaGae83UdW2aaSbaaSqaaiabdsgaKnaaBaaameaacqWGRbWAaeqaaaWcbeaaaeaacqWGRbWAaeqaniabg+GivdGccqGGSaalaaa@5482@

which differs from (3) in that it is a *density *and because the dummy event rates, λdk
 MathType@MTEF@5@5@+=feaafiart1ev1aaatCvAUfKttLearuWrP9MDH5MBPbIqV92AaeXatLxBI9gBaebbnrfifHhDYfgasaacH8akY=wiFfYdH8Gipec8Eeeu0xXdbba9frFj0=OqFfea0dXdd9vqai=hGuQ8kuc9pgc9s8qqaq=dirpe0xb9q8qiLsFr0=vr0=vr0dc8meaabaqaciaacaGaaeqabaqabeGadaaakeaaiiGacqWF7oaBdaWgaaWcbaGaemizaq2aaSbaaWqaaiabdUgaRbqabaaaleqaaaaa@317B@, now depend on the continuous breakpoint positions.

An earlier version of our software, implementing this method of using distance information, but ignoring tract-lengths, was used in a comparative analysis of *Arabidopsis thaliana*, *Arabidopsis lyrata *and *Capsella *[13].

#### Inversion tract lengths

We are interested in investigating how the rate at which inversions occur depends on the inversion tract length, i.e., the distance between inversion breakpoints. To make this question more precise, we note that if the two breakpoints defining an inversion are distributed uniformly and independently along a chromosome, then the tract length, *τ *≡ |*x*_2 _- *x*_1_|, is distributed as *p*(*τ*) ∝ (ℓ - *τ*), 0 <*τ *< ℓ, and the mean tract length is ℓ/3. Now let us consider a joint distribution of the breakpoints which falls exponentially with tract length, i.e., of the form p(x1,x2)∝e−β|x2−x1|
 MathType@MTEF@5@5@+=feaafiart1ev1aaatCvAUfKttLearuWrP9MDH5MBPbIqV92AaeXatLxBI9gBaebbnrfifHhDYfgasaacH8akY=wiFfYdH8Gipec8Eeeu0xXdbba9frFj0=OqFfea0dXdd9vqai=hGuQ8kuc9pgc9s8qqaq=dirpe0xb9q8qiLsFr0=vr0=vr0dc8meaabaqaciaacaGaaeqabaqabeGadaaakeaacqWGWbaCcqGGOaakcqWG4baEdaWgaaWcbaGaeGymaedabeaakiabcYcaSiabdIha4naaBaaaleaacqaIYaGmaeqaaOGaeiykaKIaeyyhIuRaemyzau2aaWbaaSqabeaacqGHsisliiGacqWFYoGycqGG8baFcqWG4baEdaWgaaadbaGaeGOmaidabeaaliabgkHiTiabdIha4naaBaaameaacqaIXaqmaeqaaSGaeiiFaWhaaaaa@44AD@. With this distribution of breakpoints, the tract-length distribution is *p*(*τ*) ∝ (ℓ - *τ*)*e*^-*βτ*^, and the mean tract length is

τ¯=e−βℓ(2+βℓ)+βℓ−2β(e−βℓ+βℓ−1).
 MathType@MTEF@5@5@+=feaafiart1ev1aaatCvAUfKttLearuWrP9MDH5MBPbIqV92AaeXatLxBI9gBaebbnrfifHhDYfgasaacH8akY=wiFfYdH8Gipec8Eeeu0xXdbba9frFj0=OqFfea0dXdd9vqai=hGuQ8kuc9pgc9s8qqaq=dirpe0xb9q8qiLsFr0=vr0=vr0dc8meaabaqaciaacaGaaeqabaqabeGadaaakeaaiiGacuWFepaDgaqeaiabg2da9maalaaabaGaemyzau2aaWbaaSqabeaacqGHsislcqWFYoGycqWItecBaaGccqGGOaakcqaIYaGmcqGHRaWkcqWFYoGycqWItecBcqGGPaqkcqGHRaWkcqWFYoGycqWItecBcqGHsislcqaIYaGmaeaacqWFYoGycqGGOaakcqWGLbqzdaahaaWcbeqaaiabgkHiTiab=j7aIjabloriSbaakiabgUcaRiab=j7aIjabloriSjabgkHiTiabigdaXiabcMcaPaaacqGGUaGlaaa@4FCD@

Now, defining

A(ℓ,β)=∫0ℓ∫0x2e−β|x2−x1|dx1dx2=ℓ2(e−βℓ+βℓ−1)/(βℓ)2,
 MathType@MTEF@5@5@+=feaafiart1ev1aaatCvAUfKttLearuWrP9MDH5MBPbIqV92AaeXatLxBI9gBaebbnrfifHhDYfgasaacH8akY=wiFfYdH8Gipec8Eeeu0xXdbba9frFj0=OqFfea0dXdd9vqai=hGuQ8kuc9pgc9s8qqaq=dirpe0xb9q8qiLsFr0=vr0=vr0dc8meaabaqaciaacaGaaeqabaqabeGadaaakeaacqWGbbqqcqGGOaakcqWItecBcqGGSaaliiGacqWFYoGycqGGPaqkcqGH9aqpdaWdXaqaamaapedabaGaemyzau2aaWbaaSqabeaacqGHsislcqWFYoGycqGG8baFcqWG4baEdaWgaaadbaGaeGOmaidabeaaliabgkHiTiabdIha4naaBaaameaacqaIXaqmaeqaaSGaeiiFaWhaaOGaemizaqMaemiEaG3aaSbaaSqaaiabigdaXaqabaGccqWGKbazcqWG4baEdaWgaaWcbaGaeGOmaidabeaaaeaacqaIWaamaeaacqWG4baEdaWgaaadbaGaeGOmaidabeaaa0Gaey4kIipaaSqaaiabicdaWaqaaiabloriSbqdcqGHRiI8aOGaeyypa0JaeS4eHW2aaWbaaSqabeaacqaIYaGmaaGccqGGOaakcqWGLbqzdaahaaWcbeqaaiabgkHiTiab=j7aIjabloriSbaakiabgUcaRiab=j7aIjabloriSjabgkHiTiabigdaXiabcMcaPiabc+caViabcIcaOiab=j7aIjabloriSjabcMcaPmaaCaaaleqabaGaeGOmaidaaOGaeiilaWcaaa@6A8A@

the total rate of inversions is *λ*_*I*_*A*(ℓ, *β*) including inversions of segments containing no markers. Excluding these and summing over chromosomes:

ΛI=λI∑j=1C(A(ℓj,β)−∑i=0mjA(sij,β)).
 MathType@MTEF@5@5@+=feaafiart1ev1aaatCvAUfKttLearuWrP9MDH5MBPbIqV92AaeXatLxBI9gBaebbnrfifHhDYfgasaacH8akY=wiFfYdH8Gipec8Eeeu0xXdbba9frFj0=OqFfea0dXdd9vqai=hGuQ8kuc9pgc9s8qqaq=dirpe0xb9q8qiLsFr0=vr0=vr0dc8meaabaqaciaacaGaaeqabaqabeGadaaakeaacqqHBoatdaWgaaWcbaGaemysaKeabeaakiabg2da9GGaciab=T7aSnaaBaaaleaacqWGjbqsaeqaaOWaaabCaeaadaqadaqaaiabdgeabjabcIcaOiabloriSnaaBaaaleaacqWGQbGAaeqaaOGaeiilaWIae8NSdiMaeiykaKIaeyOeI0YaaabCaeaacqWGbbqqcqGGOaakcqWGZbWCdaWgaaWcbaGaemyAaKMaemOAaOgabeaakiabcYcaSiab=j7aIjabcMcaPaWcbaGaemyAaKMaeyypa0JaeGimaadabaGaemyBa02aaSbaaWqaaiabdQgaQbqabaaaniabggHiLdaakiaawIcacaGLPaaaaSqaaiabdQgaQjabg2da9iabigdaXaqaaiabdoeadbqdcqGHris5aOGaeiOla4caaa@57A7@

We will analyze unsigned data, i.e., we use the positions of the markers but not the orientations of individual markers. In this case inversions containing one marker are undetectable. However, since finding the shortest inversion path is hard for the unsigned case, we study the unsigned problem by working with signed arrangements of markers, and sampling from the set of all (signed) paths consistent with the unsigned data, as described in [6]. This means our paths may include one or more 1-marker inversions.

#### Paracentric and pericentric inversions

We want to allow pericentric and paracentric inversions to occur at different rates. Under the uniform independent breakpoint distribution assumption the mean tract length of pericentric inversions is ℓ¯pe
 MathType@MTEF@5@5@+=feaafiart1ev1aaatCvAUfKttLearuWrP9MDH5MBPbIqV92AaeXatLxBI9gBaebbnrfifHhDYfgasaacH8akY=wiFfYdH8Gipec8Eeeu0xXdbba9frFj0=OqFfea0dXdd9vqai=hGuQ8kuc9pgc9s8qqaq=dirpe0xb9q8qiLsFr0=vr0=vr0dc8meaabaqaciaacaGaaeqabaqabeGadaaakeaacuWItecBgaqeamaaBaaaleaacqWGWbaCcqWGLbqzaeqaaaaa@30DD@ = ℓ/2 independent of the centromere position. For paracentric inversions on a chromosome arm of length *q *the mean tract length is *q*/3 and the inversion rate is proportional to *q*^2^. For a chromosome with arm lengths *ξ*ℓ and (1 - *ξ*)ℓ, this leads to a mean paracentric tract length of

ℓ¯pa=(ξ2+(1−ξ)3ξ2+(1−ξ)2)ℓ/3.
 MathType@MTEF@5@5@+=feaafiart1ev1aaatCvAUfKttLearuWrP9MDH5MBPbIqV92AaeXatLxBI9gBaebbnrfifHhDYfgasaacH8akY=wiFfYdH8Gipec8Eeeu0xXdbba9frFj0=OqFfea0dXdd9vqai=hGuQ8kuc9pgc9s8qqaq=dirpe0xb9q8qiLsFr0=vr0=vr0dc8meaabaqaciaacaGaaeqabaqabeGadaaakeaacuWItecBgaqeamaaBaaaleaacqWGWbaCcqWGHbqyaeqaaOGaeyypa0ZaaeWaaeaadaWcaaqaaGGaciab=57a4naaCaaaleqabaGaeGOmaidaaOGaey4kaSIaeiikaGIaeGymaeJaeyOeI0Iae8NVdGNaeiykaKYaaWbaaSqabeaacqaIZaWmaaaakeaacqWF+oaEdaahaaWcbeqaaiabikdaYaaakiabgUcaRiabcIcaOiabigdaXiabgkHiTiab=57a4jabcMcaPmaaCaaaleqabaGaeGOmaidaaaaaaOGaayjkaiaawMcaaiabloriSjabc+caViabiodaZiabc6caUaaa@4BF9@

Depending on *ξ*, ℓ¯pa
 MathType@MTEF@5@5@+=feaafiart1ev1aaatCvAUfKttLearuWrP9MDH5MBPbIqV92AaeXatLxBI9gBaebbnrfifHhDYfgasaacH8akY=wiFfYdH8Gipec8Eeeu0xXdbba9frFj0=OqFfea0dXdd9vqai=hGuQ8kuc9pgc9s8qqaq=dirpe0xb9q8qiLsFr0=vr0=vr0dc8meaabaqaciaacaGaaeqabaqabeGadaaakeaacuWItecBgaqeamaaBaaaleaacqWGWbaCcqWGHbqyaeqaaaaa@30D5@ lies between ℓ/3 and ℓ/6, so for any centromere position ℓ¯pa
 MathType@MTEF@5@5@+=feaafiart1ev1aaatCvAUfKttLearuWrP9MDH5MBPbIqV92AaeXatLxBI9gBaebbnrfifHhDYfgasaacH8akY=wiFfYdH8Gipec8Eeeu0xXdbba9frFj0=OqFfea0dXdd9vqai=hGuQ8kuc9pgc9s8qqaq=dirpe0xb9q8qiLsFr0=vr0=vr0dc8meaabaqaciaacaGaaeqabaqabeGadaaakeaacuWItecBgaqeamaaBaaaleaacqWGWbaCcqWGHbqyaeqaaaaa@30D5@ <ℓ¯pe
 MathType@MTEF@5@5@+=feaafiart1ev1aaatCvAUfKttLearuWrP9MDH5MBPbIqV92AaeXatLxBI9gBaebbnrfifHhDYfgasaacH8akY=wiFfYdH8Gipec8Eeeu0xXdbba9frFj0=OqFfea0dXdd9vqai=hGuQ8kuc9pgc9s8qqaq=dirpe0xb9q8qiLsFr0=vr0=vr0dc8meaabaqaciaacaGaaeqabaqabeGadaaakeaacuWItecBgaqeamaaBaaaleaacqWGWbaCcqWGLbqzaeqaaaaa@30DD@. This means that either a tract-length dependent effect (*β *> 0), or an effect which distinguishes only between paracentric and pericentric inversions, can have the effect of suppressing longer tract-length inversions. In order to know whether there is a tract-length effect independent of a possible paracentric/pericentric effect, we keep track of the two kinds of inversions separately, and assume a tract-length dependent paracentric rate

Λpa=λpa∑j=12C(A(ℓj,β)−∑i=0mjA(sij,β)),
 MathType@MTEF@5@5@+=feaafiart1ev1aaatCvAUfKttLearuWrP9MDH5MBPbIqV92AaeXatLxBI9gBaebbnrfifHhDYfgasaacH8akY=wiFfYdH8Gipec8Eeeu0xXdbba9frFj0=OqFfea0dXdd9vqai=hGuQ8kuc9pgc9s8qqaq=dirpe0xb9q8qiLsFr0=vr0=vr0dc8meaabaqaciaacaGaaeqabaqabeGadaaakeaacqqHBoatdaWgaaWcbaGaemiCaaNaemyyaegabeaakiabg2da9GGaciab=T7aSnaaBaaaleaacqWGWbaCcqWGHbqyaeqaaOWaaabCaeaadaqadaqaaiabdgeabjabcIcaOiabloriSnaaBaaaleaacqWGQbGAaeqaaOGaeiilaWIae8NSdiMaeiykaKIaeyOeI0YaaabCaeaacqWGbbqqcqGGOaakcqWGZbWCdaWgaaWcbaGaemyAaKMaemOAaOgabeaakiabcYcaSiab=j7aIjabcMcaPaWcbaGaemyAaKMaeyypa0JaeGimaadabaGaemyBa02aaSbaaWqaaiabdQgaQbqabaaaniabggHiLdaakiaawIcacaGLPaaaaSqaaiabdQgaQjabg2da9iabigdaXaqaaiabikdaYiabdoeadbqdcqGHris5aOGaeiilaWcaaa@5BC7@

where the sums are now over chromosome arms and ℓ_*j *_and *m*_*j *_are the length and number of markers for the *j*^*th *^arm. We assume a tract-length independent pericentric rate

Λpe=λpe∑j=1C(ℓ2j−1ℓ2j),
 MathType@MTEF@5@5@+=feaafiart1ev1aaatCvAUfKttLearuWrP9MDH5MBPbIqV92AaeXatLxBI9gBaebbnrfifHhDYfgasaacH8akY=wiFfYdH8Gipec8Eeeu0xXdbba9frFj0=OqFfea0dXdd9vqai=hGuQ8kuc9pgc9s8qqaq=dirpe0xb9q8qiLsFr0=vr0=vr0dc8meaabaqaciaacaGaaeqabaqabeGadaaakeaacqqHBoatdaWgaaWcbaGaemiCaaNaemyzaugabeaakiabg2da9GGaciab=T7aSnaaBaaaleaacqWGWbaCcqWGLbqzaeqaaOWaaabCaeaacqGGOaakcqWItecBdaWgaaWcbaGaeGOmaiJaemOAaOMaeyOeI0IaeGymaedabeaakiabloriSnaaBaaaleaacqaIYaGmcqWGQbGAaeqaaOGaeiykaKcaleaacqWGQbGAcqGH9aqpcqaIXaqmaeaacqWGdbWqa0GaeyyeIuoakiabcYcaSaaa@494F@

where here ℓ_2*j *- 1 _and ℓ_2*j *_are the lengths of the two arms of chromosome *j*.

Now that we distinguish between paracentric and pericentric inversions and allow for a tract-length dependent rate, (5) becomes

p(X|λpa,λpe,λT,β)=e−ΛL!λpaLpaλpeLpeλTLT∏kλdke−β∑lτl,
 MathType@MTEF@5@5@+=feaafiart1ev1aaatCvAUfKttLearuWrP9MDH5MBPbIqV92AaeXatLxBI9gBaebbnrfifHhDYfgasaacH8akY=wiFfYdH8Gipec8Eeeu0xXdbba9frFj0=OqFfea0dXdd9vqai=hGuQ8kuc9pgc9s8qqaq=dirpe0xb9q8qiLsFr0=vr0=vr0dc8meaabaqaciaacaGaaeqabaqabeGadaaakeaacqWGWbaCcqGGOaakcqWGybawcqGG8baFiiGacqWF7oaBdaWgaaWcbaGaemiCaaNaemyyaegabeaakiabcYcaSiab=T7aSnaaBaaaleaacqWGWbaCcqWGLbqzaeqaaOGaeiilaWIae83UdW2aaSbaaSqaaiabdsfaubqabaGccqGGSaalcqWFYoGycqGGPaqkcqGH9aqpdaWcaaqaaiabdwgaLnaaCaaaleqabaGaeyOeI0Iaeu4MdWeaaaGcbaGaemitaWKaeiyiaecaaiab=T7aSnaaDaaaleaacqWGWbaCcqWGHbqyaeaacqWGmbatdaWgaaadbaGaemiCaaNaemyyaegabeaaaaGccqWF7oaBdaqhaaWcbaGaemiCaaNaemyzaugabaGaemitaW0aaSbaaWqaaiabdchaWjabdwgaLbqabaaaaOGae83UdW2aa0baaSqaaiabdsfaubqaaiabdYeamnaaBaaameaacqWGubavaeqaaaaakmaarafabaGae83UdW2aaSbaaSqaaiabdsgaKnaaBaaameaacqWGRbWAaeqaaaWcbeaakiabdwgaLnaaCaaaleqabaGaeyOeI0Iae8NSdi2aaabeaeaacqWFepaDdaWgaaadbaGaemiBaWgabeaaaeaacqWGSbaBaeqaoiabggHiLdaaaaWcbaGaem4AaSgabeqdcqGHpis1aOGaeiilaWcaaa@749C@

where now Λ(*λ*_*pa*_, *λ*_*pe*_, *λ*_*T*_) > Λ_*real *_= Λ_*pa *_+ Λ_*pe *_+ Λ_*T*_, and *λ*_*d *_= Λ - Λ_*real *_as before, and *τ*_*l *_is the tract length of the *l*^*th *^paracentric inversion.

Now we can write down the posterior probability:

p(X,λpa,λpe,λT,β|D)∝P(D|X)e−ΛL!λpaLpaλpeLpeλTLT×∏kλdke−β∑lτlp(λpa)p(λpe)p(λT)p(β).
 MathType@MTEF@5@5@+=feaafiart1ev1aaatCvAUfKttLearuWrP9MDH5MBPbIqV92AaeXatLxBI9gBaebbnrfifHhDYfgasaacH8akY=wiFfYdH8Gipec8Eeeu0xXdbba9frFj0=OqFfea0dXdd9vqai=hGuQ8kuc9pgc9s8qqaq=dirpe0xb9q8qiLsFr0=vr0=vr0dc8meaabaqaciaacaGaaeqabaqabeGadaaakeaafaqabeGabaaabaGaemiCaaNaeiikaGIaemiwaGLaeiilaWccciGae83UdW2aaSbaaSqaaiabdchaWjabdggaHbqabaGccqGGSaalcqWF7oaBdaWgaaWcbaGaemiCaaNaemyzaugabeaakiabcYcaSiab=T7aSnaaBaaaleaacqWGubavaeqaaOGaeiilaWIae8NSdiMaeiiFaWNaemiraqKaeiykaKIaeyyhIuRaemiuaaLaeiikaGIaemiraqKaeiiFaWNaemiwaGLaeiykaKYaaSaaaeaacqWGLbqzdaahaaWcbeqaaiabgkHiTiabfU5ambaaaOqaaiabdYeamjabcgcaHaaacqWF7oaBdaqhaaWcbaGaemiCaaNaemyyaegabaGaemitaW0aaSbaaWqaaiabdchaWjabdggaHbqabaaaaOGae83UdW2aa0baaSqaaiabdchaWjabdwgaLbqaaiabdYeamnaaBaaameaacqWGWbaCcqWGLbqzaeqaaaaakiab=T7aSnaaDaaaleaacqWGubavaeaacqWGmbatdaWgaaadbaGaemivaqfabeaaaaaakeaacqGHxdaTdaqeqbqaaiab=T7aSnaaBaaaleaacqWGKbazdaWgaaadbaGaem4AaSgabeaaaSqabaGccqWGLbqzdaahaaWcbeqaaiabgkHiTiab=j7aInaaqababaGae8hXdq3aaSbaaWqaaiabdYgaSbqabaaabaGaemiBaWgabeGdcqGHris5aaaakiabdchaWjabcIcaOiab=T7aSnaaBaaaleaacqWGWbaCcqWGHbqyaeqaaOGaeiykaKIaemiCaaNaeiikaGIae83UdW2aaSbaaSqaaiabdchaWjabdwgaLbqabaGccqGGPaqkcqWGWbaCcqGGOaakcqWF7oaBdaWgaaWcbaGaemivaqfabeaakiabcMcaPiabdchaWjabcIcaOiab=j7aIjabcMcaPiabc6caUaWcbaGaem4AaSgabeqdcqGHpis1aaaaaaa@9A2C@

The *λ*'s priors are all uniform between 0 and *λ*_*max *_and zero elsewhere. We assume *β *≥ 0 with a uniform prior. We assume that chromosomes always have exactly one centromere. In the computer code the breakpoint graph only considers marker-marker adjacencies, not marker-centromere adjacencies, and this means the way proposed rearrangement paths are constructed does not guarantee that centromeres end up in the right place. The centromeres are just passively carried along by the inversions and rearrangements dictated by the breakpoint graph. If the centromeres do not end up in the right place, the proposed path is rejected in the MCMC updating step, leading to loss of efficiency, but not loss of correctness. If the centromere lies within a region of conserved marker order its probability of ending up in the right place will typically be high, but if it lies between conserved regions this probability may be quite low, contributing to a low MCMC acceptance probability.

### Data processing

We chose *D. yakuba *to compare with *D. melanogaster*. This choice was dictated by the need to have sufficiently many inversions that the biological problem is interesting, but not so many inversions that computational complexity becomes too large. We started with chained and netted alignments as described in [14]. We used the "net" file droYak2.dm2.net.gz, downloaded from the UC Santa Cruz website. This file contains information on chained alignments ('chains"), organized into hierarchies called "nets". These alignments are based on the Nov. 2005 WUSTL version 2.0 *D. yakuba *assembly and the Apr. 2004, BDGP v. 4/DHGP v. 3.2 *D. melanogaster *assembly.

**Figure 8:**
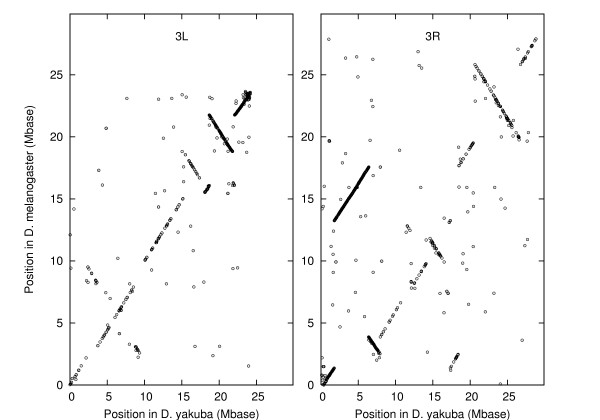
Position in *D. melanogaster *vs. position in *D. yakuba *for chains identified as on the same arm of chromosome 3 in both species.

Figure 8 shows the position in *D. melanogaster *of each chain, plotted versus it's position in *D. yakuba*. The figure shows the 982 chains located on chromosome arm 3*L *in both species and the 1,322 chains located on 3*R *in both species. Many points lie along lines with slopes close to ± 1, as expected for markers rearranged by inversions and translocations. There are, however, many other points scattered about, requiring further processing. First, chains not labeled in the net file as of type "syn" (i.e., syntenic) are eliminated. The chains left after some additional processing will be the markers used by the analysis program; from here on we refer to markers rather than chains.

Remaining markers are further processed by defining blocks within which adjacency is conserved. Two markers which are adjacent in both species are in the same block; if adjacent in just one species they are in different blocks. Blocks containing only a single marker are discarded, and blocks shorter than a minimum length are replaced by a single marker at the block's average position. This procedure is then repeated and the number of blocks may decrease, both directly because of discarding one-marker blocks, and also because when a block is discarded, or when a block is shortened to one marker, neighboring blocks will often join into one block. This procedure is repeated several times while the minimum block length is gradually increased from 100 bases to some final value *L*_*min*_. Thus, a long block can emerge from a set of short blocks as some are eliminated and others join together. In some cases a block which ideally would be retained and incorporated into a long block may be lost during this process, if it is shorter than *L*_*min *_and doesn't join another block soon enough. This can cause gaps in the spacing of markers on the resulting long block or the shortening of the block at an end. Neither of these is a big problem, although shortening at the ends of blocks means breakpoints are less well localized. The set of blocks generated is insensitive to *L*_*min *_over a broad range: for our data, any value of *L*_*mim *_between 25 kilobases and 115 kilobases gives the set of blocks that we analyzed.

Finally, markers are thinned from blocks containing many markers, until no block has more than 8 markers. Markers at the ends of blocks are kept, and the thinning of the others is done so as get a fairly even spacing. This reduces the time and memory requirements of the program, while having little effect on posterior distributions, according to our studies.

Applied to chromosomes *X*, 2, and 3, this procedure gives the 388 markers in 56 blocks shown in figures 1, 2, and 3.

## Authors' contributions

RN had the idea of using MCMC methods to study chromosomal rearrangements and of using distance information to study tract lengths. RD contributed key mathematical techniques. TY wrote the MCMC code, analyzed the data, and wrote most of the manuscript. RN wrote parts of the manuscript and all authors participated in revising it. All authors have read and approved the final manuscript.
